# Pre-clinical Models of Metastasis in Pancreatic Cancer

**DOI:** 10.3389/fcell.2021.748631

**Published:** 2021-10-27

**Authors:** Maria Miquel, Shuman Zhang, Christian Pilarsky

**Affiliations:** ^1^Department of Surgery, University Hospital, Erlangen, Germany; ^2^Friedrich-Alexander-Universität Erlangen-Nürnberg, Erlangen, Germany

**Keywords:** metastasis, pancreatic cancer, organoids, metastasis models, PDAC – pancreatic ductal adenocarcinoma, GEMMs

## Abstract

Pancreatic ductal adenocarcinoma (PDAC) is a hostile solid malignancy coupled with an extremely high mortality rate. Metastatic disease is already found in most patients at the time of diagnosis, resulting in a 5-year survival rate below 5%. Improved comprehension of the mechanisms leading to metastasis is pivotal for the development of new targeted therapies. A key field to be improved are modeling strategies applied in assessing cancer progression, since traditional platforms fail in recapitulating the complexity of PDAC. Consequently, there is a compelling demand for new preclinical models that mirror tumor progression incorporating the pressure of the immune system, tumor microenvironment, as well as molecular aspects of PDAC. We suggest the incorporation of 3D organoids derived from genetically engineered mouse models or patients as promising new tools capable to transform PDAC pre-clinical modeling and access new frontiers in personalized medicine.

## Introduction

Pancreatic ductal adenocarcinoma (PDAC) has the worst 5-year relative survival rate in comparison to all other solid tumors and has been prognosed to become the second leading cause of cancer-related mortality in the United States by 2030 after lung cancer (Chu et al., 2017; McGuigan et al., 2018). More than 90% of pancreatic cancers are exocrine tumors, being the most frequent type, PDAC. Other tumors like neuroendocrine tumors (PNET) are often indolent and treatable (Antonello et al., 2009). The poor outcome is correlated to late diagnosis, a result of non-specific symptoms, poor specificity of tumor markers, and non-accessible sites for routine palpation. Further, the PDAC is associated with a high capacity of metastatic dissemination to adjacent organs already in small tumor sizes. Common sites of dissemination are the liver, with metastases present in 76–80% of patients, peritoneum (48%) and the lungs (45%; Yachida et al., 2012). Even though surgical resection of the primary tumor is the only treatment with curative intention, 85–90% of patients are not eligible due to the systemic nature of the disease and a lack of early diagnosis. Even in the less than 20% operable cases, where the primary tumor has been completely removed (R0) and no manifestation of metastasis at resection, 75% of the patients will die of metastatic relapse in then 5 years after being operated (Chu et al., 2017; McGuigan et al., 2018).

### Genetics

Pancreatic ductal adenocarcinoma is a complex genetic disease, mainly determined by oncogenic activation of Kirsten rat sarcoma virus (KRAS) and mutations in tumor suppressor genes such Cyclin Dependent Kinase Inhibitor 2A (CDKN2A), Transformation Related Protein 53 (TP53), Lysine Demethylase 6A (KDM6A), Breast Cancer Gene (BRCA1/2), and SMAD Family Member 4 (SMAD4; Yachida and Iacobuzio-Donahue, 2009). The signature mutations of PDAC were identified in precursor lesions namely pancreatic intraepithelial neoplasia (PanIN), mucinous cystic neoplasia (MCNs), and intraductal papillary mucinous neoplasm (IPMNs; Hruban et al., 2001). Activation of oncogenic Kras in pancreatic epithelial cells triggers initiation of PDAC in mouse models and when combined with Trp53, Cdkn2a, or Smad4 mutations PDAC progression is accelerated, recapitulating many characteristics of the human disease (Izeradjene et al., 2007; see section “Genetically engineered mouse models”).

### Subtypes

Using bulk tumor samples, separate studies identified at least two subtypes of PDAC (Collisson et al., 2011; Moffitt et al., 2015; Bailey et al., 2016; Chan-Seng-Yue et al., 2020), differentiated by markers of epithelial differentiation state, being the less differentiated subtype (“basal-like,” “squamous,” or “quasi-mesenchymal”) the one correlating with worse prognosis compared to the better differentiated subtypes (“classical” or “progenitor”; Dreyer et al., 2021a).

In primary patient-derived cell lines and bulky tumors of the various PDAC cohorts, a replication stress signature linked with the squamous subtype was identified. This is linked with functional impairments in replication of DNA and might also be utilized as biomarkers and give alternative therapeutics choices to standard care platinum chemotherapy for patients with DNA replication abnormalities (Dreyer et al., 2021b). The squamous subtype has also been defined by a distinct metabolic phenotype due to loss of genes that specify endodermal lineage, Hepatocyte Nuclear Factor 4 Alpha (HNF4A), and GATA Binding Protein 6 (GATA6). This subtype is therefore more sensitive to Glycogen synthase kinase 3 beta (GSK3β) inhibition except for a subgroup with distinct chromatin accessibility which acquires rapid drug resistance (Brunton et al., 2020).

Employing laser capture microdissection and RNA sequencing on PDAC epithelia and adjacent stroma defined two stromal subtypes differing in the immune-associated and extracellular matrix-associated processes. This study showed that across the same tumors, epithelial and stromal subtypes were partially linked [Extracellular Matrix (ECM) rich stroma was associated with Basal-like epithelium and Immune-rich stroma was found more often in association with Classical epithelia], showing potential dependence in the evolution of the tissue compartments in PDAC (Maurer et al., 2019).

Another study, based on the methylation patterns of the tumor genomes, defined two different origins of adenocarcinomas. One type of tumor is formed directly from ductal cells lining the ductal system of the pancreas, whereas the other develops from glandular cells and is less aggressive (Espinet et al., 2021).

Despite the current classification consensus, Juiz et al. showed that Basal-like and Classical cells coexist in PDAC as described by single-cell analysis on pancreatic cancer organoids derived from biopsies indicating that both subtypes can coexist in the same patient (Juiz et al., 2020).

### Chemotherapy

Even though clinical decision-making based on histopathological criteria is widely established in several cancer types, subtypes of PDAC currently do not guide treatment decisions (Collisson et al., 2019). The only treatment with curative intent is surgery, which can be preceded by a neoadjuvant treatment and followed by adjuvant therapies such as gemcitabine monotherapy. However, recurrence rates in operated patients are still high and long-term survival is limited (Bijlsma, 2021).

The current standard of care for metastatic PDAC includes highly toxic chemotherapeutic cocktails with limited specificities. Gemcitabine has become a widely used drug for advanced and metastatic PDAC since it was reported (Burris et al., 1997), despite its low influence on patient survival. There are two gold-standard combination regimens for metastatic PDAC: 5-fluorouracil/leucovorin with irinotecan and oxaliplatin (FOLFIRINOX; Conroy et al., 2011), and gemcitabine with nab-paclitaxel since 2011 (Peixoto et al., 2017). A detailed review of PDAC chemotherapy can be found elsewhere: (Zeng et al., 2019; Singh and O’Reilly, 2020). According to developments and advances in other cancer types, it is expected that improvements in PDAC treatment are likely to come from the combination of classical cytotoxics with novel targeted agents against PDAC. Important matters in hand related to new therapeutic approaches include immunotherapy, DNA damage repair strategies, targeting the stroma, as well as cancer-cell metabolism (Dreyer et al., 2021b).

Current targeted therapies in PDAC undergoing Clinical Trials are divided into three approaches (Table 1). Firstly, inhibition of dysregulated oncogenes such as KRAS, c-MYC, Neurotrophic tyrosine receptor kinase, Neuregulin 1, and related molecules. Since these options have not led to an improvement of patient survival, alternative strategies are being developed to target these oncogenes, namely modification of mutant residues by small molecules, simultaneously inhibiting multiple molecules or pathways, and RNA interference. Secondly, reactivate tumor suppressors or modulate related molecules such as TP53, CDKN2A, SMAD4, KDM6A, and BRCA1/2. In addition to genetic event-guided treatment, immunotherapies such as antibody-drug conjugates, chimeric antigen receptor T cells (CAR-T), and immune checkpoint inhibitors also indicate the potential to target tumors precisely. Nonetheless, targeted therapies have been largely unsuccessful in PDAC. Currently, the only targeted therapeutic agent approved for PDAC is Erlotinib, which only slightly prolongs survival in metastatic disease (Moore et al., 2007; Sinn et al., 2017) but showed negative results in the adjuvant setting (Bijlsma et al., 2017). A possible reason for the unsuccessful outcomes of targeted agents in PDAC is incorrect patient selection. Interestingly, when tumor samples from several of the CONKO-005 trial participants were re-analyzed, a subgroup of patients with a combination of SMAD4 loss and low Mitogen-Activated Protein Kinase 9 (MAPK9) expression benefited from the addition of Erlotinib (Hoyer et al., 2021). Therefore, not only novel targeted therapies are needed but also integration with genomic profiling along with a full understanding of the tumor microenvironment and immunology (Qian et al., 2020). It is expected that in the future, comprehensive tumor analysis should become an essential part of diagnostic routines and guide treatment choice.

**TABLE 1:** Potential therapeutic targets in PDAC undergoing Clinical Trials.

Gene alterations (Targets)	Mutation rate (% af all tumors)	Potential target	Therapeutic mechanism	Promising agents	Combination partner	Study phase	Reference Clinical Trial
KRAS	90%	EGFR	KRAS inhibition	Nimotuzumab	Gemcitabine	Phase II	OSAG101-PCS07, NCT00561990
				Afatinib	Capecitabine	Phase I	NCT02451553
				Erlotinib	Gemcitabine	Phase III	CONKO-005, DRKS00000247
			Inhibits the intracellular phosphorylation of tyrosine kinase associated EGFR	Erlotinib	Selumetinib	Phase II	NCT01222689
		KRAS G12D/G12V	Small interfering RNA	siG12D LODER	Gemcitabine + nab-Paclitaxel Folfirinox	Phase II	NCT01676259
		KRAS G12C	Small-molecule inhibitor	MRTX849 (Adagrasib)	Afatinib Pembrolizumab Cetuximab	Phase I-II	NCT03785249, NCT04330664
				AMG 510 (sotorasib)	anti PD-1/L1	Phase I-II	NCT03600883
		PI3K-PLK1	Disrupts RAF and PI3K family binding to RAS	ON 01910.Na (Rigosertib)	Gemcitabine	Phase III	NCT01360853
			Allosteric AKT inhibitor	MK-2206	Selumetinib	Phase II	NCT01658943
CDKN2A	60%	CDK4-6	Cell cycle blockade via pRb	Ribociclib	Trametinib	Phase I-II	NCT02703571
				LY2835219 (Abemaciclib)	LY3023414 Gemcitabine Capecitabine	Phase II	NCT02981342
SMAD4	50%	TGFβ	Inhibits signaling through TGFβ-I receptor	Galunisertib	Gemcitabine	Phase I-II	NCT01373164
BRCA1/2	5%	PARP	PARP inhibition	Olaparib		Phase III	POLO trial: NCT02184195
MSIH/dMMR	1%	PD1	PD-1/PD-L1Immune checkpoint inhibition	Pembrolizumab	BL-8040 Onivyde/5-FU/LV	Phase II	KEYNOTE-158, NCT02628067 COMBAT, NCT02826486
NRG1	0.5%	ERBB3	Targets the HER2:HER3 heterodimer	MCLA128 (zenocutuzumab)		Phase I-II	NCT02912949
NTRK	0.3%	TRK	TRK inhibition	Entrectinib Larotrectinib		Phase I-II trials	NCT02122913 NCT02097810 NCT02568267
			NTRK mutations inhibition	Selitrectinib Repotrectinib		Phase I/II trials	NCT03215511 NCT03093116
c-MET	0.3%	MET	ALK/ROS inhibitor	Crizotinib		Phase I/II trials	NCT04693468
CXCR4	0.3%	CXCR4	Chemokine receptor inhibitor	BL-8040 (Motixafortide)	Pembrolizumab Onivyde/5-FU/LV	Phase II	COMBAT, NCT02826486

*All the Clinical Trials described include patients with locally advanced or metastatic disease except the CONKO-005 trial which is focused on R0-resected pancreatic cancer.*

Since most PDAC patients succumb due to metastatic cancer, this accentuates the crucial need to develop novel therapies that target, not only the primary tumor, but also the vulnerabilities of metastatic cells (Singh and O’Reilly, 2020). The last results of the COMBAT/KEYNOTE-202 Trial showed that Triple combination of motixafortide, pembrolizumab and chemotherapy are safe, well tolerated and showed signs of efficacy in a population with poor prognosis and aggressive PDAC (Bockorny et al., 2021). Promising new anticancer compounds are tested pre-clinically into *in vitro* and *in vivo* models. However, 90% of those fail when moving to clinical trials (Lai et al., 2019), showing the need for more consistent and representative models for drug testing, recapitulating better genetics, immunology, physiology, and metabolism from the human disease. Recent studies have highlighted the use of patient-derived organoids (PDOs) as a personalized model suitable for High throughput screening (HTS) which might help overcome some of the current model limitations (Tiriac et al., 2019).

### Stroma

Pancreatic ductal adenocarcinoma is characterized by dense desmoplasia, which can compose up to 80% of the whole tumor volume and low tumor cellularity, while metastases in the liver have less stroma and more tumor cellularity than primary tumors resulting in less overall survival (Rucki, 2014).

A complex network of inflammatory cells, fibroblasts, ECM and vasculature maintain tissue homeostasis in the stroma of normal epithelial tissues. In Contrast, around pancreatic cancer tissues, neoplastic cells corrupt the stroma to form a tumor-promoting environment which, at the same turn, promotes cancer cell proliferation and migration, and provides a reservoir for growth factors and cytokines.

The three dominant entities in the PDAC stroma are the vasculature, ECM, and cancer-associated fibroblasts (CAFs). Molecular subtypes of pancreatic CAFs have been described (Öhlund et al., 2017), most remarkably myofibroblastic CAFs and inflammatory CAFs, which have been speculated to participate in active crosstalk with cancer cells and pro-tumor and antitumor properties, respectively. Tumor progression is linked with disruption of the basement membrane integrity and desmoplastic reaction with enhanced production of type I collagen (Öhlund et al., 2009; Shields et al., 2012). Moffitt et al. (2015) by digitally separating tumor, normal and stromal gene expression, defined “normal” and “activated” stromal subtypes, which are independently prognostic of PDAC. Using a Hedgehog inhibitor, the decrement of stroma was beneficial in mouse models due to the blockade of stromal growth factors and elimination of the barrier for therapeutic delivery (Olive et al., 2009). Despite those observations, several strategies to target the ECM have been pursued in the last years but have so far failed to show an increase in patient survival (Hosein et al., 2020; Tomás-Bort et al., 2020). Experimentally manipulating stromal matrix content led to lower tissue stiffness, and increased tumor growth, resulting in decreased overall survival. Similarly, a multitude of anti-angiogenesis agents have been unsuccessful in late-stage clinical trials of PDAC probably due to hypovascularity (Hosein et al., 2020).

Since desmoplasia promotes hypovascularity and immunosuppression it results in a hypoxic environment due to limited oxygen diffusion through the tumor (Tuveson and Neoptolemos, 2012). In-depth research has shown that hypoxia modulates tumor biology promoting malignancy through hypoxia inducible factors (HIFs), which should be considered for targeted therapy (Shah et al., 2020). Hypoxia signaling also affects stromal cells, enhancing activation of macrophages, CAFs, stem cells, and secretion of specific ECM factors to produce widespread stroma of PDAC (Keith et al., 2012).

### Metastasis

**FIGURE 1:**
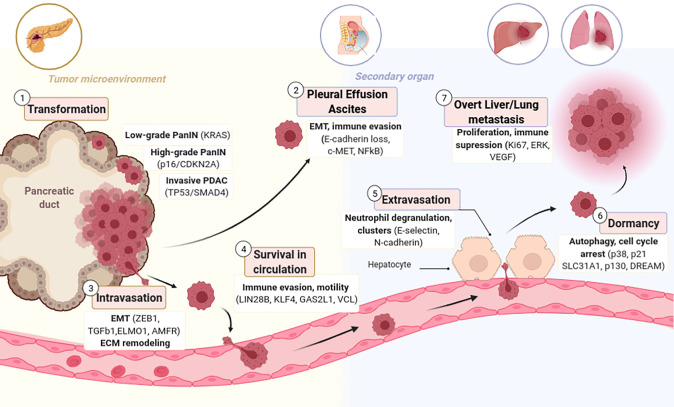
Stages of Metastatic Progression with candidate genes responsible per each stage. (1) Normal epithelial pancreatic ductal cells acquire an aggressive phenotype through serial mutations that transform them firstly to PanIN and lately to PDAC. (2) Transformed cells are capable of detaching and colonizing the Peritoneum forming Ascites or Pleural Effusion. (3) PDAC cells have enhanced motility due to EMT that allows them to invade blood or lymphatic vessels. (4) CTCs in circulation are abundant, but only few survive this pressure. (5) Several CTCs have tropism for the pre-metastatic niche (PMN) and are able to extravasate to a secondary organ where they might remain dormant (6) for several years and eventually relapse and form overt metastasis (7).

Metastasis is characterized by a sequential process initiated by the invasion of carcinoma cells into the basement membrane into the neighboring stroma, followed by invasion and survival into circulation (blood, lymph), extravasation into the parenchyma of distant tissues, and lastly by the reestablishment of foci of neoplastic cells at remote sites, even after a period of dormancy (Massagué and Obenauf, 2016; Massagué et al., 2017; Figure 1). Accordingly, a central step in the metastatic process is the gain of migratory and invasive phenotype. This demands pancreatic cancer cells to switch many of their epithelial characteristics for mesenchymal traits through a cellular program named epithelial to mesenchymal transition (EMT). The opposite process, mesenchymal to epithelial transition (MET), happens when colonies are re-established at the secondary organ (Beuran et al., 2015).

The decisive promoters of PDAC metastasis are not yet sufficiently understood, notably since the genetic composition of most metastases closely resembles the one of the complementary primary tumors (Campbell et al., 2010; Yachida et al., 2010; Makohon-Moore et al., 2017). The most studied drivers of metastasis in PDAC are: TP53 (Morton et al., 2010), SMAD4 (Ahmed et al., 2017), aberrant Wnt signaling (Yu et al., 2012), and aberrant ECM gene expression (Harris et al., 2017). Also, reduced expression in Liver Kinase B1 (LKB1) is correlated with liver metastasis, vascular invasion and thus, worse overall survival (Yang et al., 2015, 1). It has been suggested that Transforming growth factor beta (TGF-β) promotes invasion and migration via the initiation of EMT (Padua and Massagué, 2009; Aiello et al., 2017). Molecular perturbations coupled with this transition combine the loss of epithelial markers such as cytokeratins, E-cadherin, occludin, and claudin, with the gain of mesenchymal markers namely N-cadherin, fibronectin and vimentin (Maier et al., 2010), loss of cell-cell contacts and polarity leading to the gain of a mesenchymal migratory behavior (Thiery, 2002). Further, cells that have transitioned to the mesenchymal state embrace a spindle-like shape instead of a columnar one and have elevated invasiveness, migratory capacity, enhanced resistance to apoptosis and increased production of ECM factors (Rhim et al., 2012). It has been described that the PDAC EMT program is defined by an intermediate cell state “partial EMT” consisting of the maintenance of an epithelial program only at the protein level (Jolly, 2015). Partial EMT cells can migrate individually or as clusters while complete EMT cells mainly migrate in isolation (Duda et al., 2010; Grigore et al., 2016; Saitoh, 2018). The various mechanisms of dissemination (single cancer cells or clusters) seem to affect the metastatic capacity of cancer cells, since single cells do not have such a high metastatic capacity as tumor clusters (Friedl et al., 2012; Cheung and Ewald, 2016). Tumor clusters can be heterogeneous and integrated of stromal cells, such as CAFs, co-migrating with cancer cells to remote sites (Wang et al., 2016). Overall, in PDAC, the EMT program has been proved to enhance tumor-initiating potential (Rasheed et al., 2010) and drug resistance (Zhou et al., 2017).

When epithelial cells undergo EMT and enter circulation become circulating tumor cells (CTCs; Figure 1). CTCs isolated from patient blood express a cell motility gene signature consisting of upregulation of EMT and motility inducing genes such as Vinculin, Engulfment and cell motility protein 1, Autocrine Motility Factor Receptor, TFGß1 or p38 (Sergeant et al., 2012). CTCs necessarily contain the precursors of distant metastatic foci; thus, they may be characterized to identify drivers of dissemination, correlate gene set metastatic signatures, and develop targeted therapies to the “seeds” of metastasis (Franses et al., 2020). Using primarily CTCs collected from Genetically engineered mouse models (GEMMs), Growth Arrest Specific 2 Like 1 has been identified as a potential biomarker of CTCs in PDAC (Zhu et al., 2020). Contrary to the previous acceptance that PanINs are not able to undergo EMT, it has been reported that also low-grade PanINs, harboring only activating KRAS mutations, show indication of cells that have exfoliated and become CTCs expressing CD24 and CD44 (Rhim et al., 2012). Inflammation enhances the amount of circulating pancreatic preneoplastic cells, supporting the association between inflammation and PDAC (Mazur and Siveke, 2012). Consistently, treatment with anti-inflammatory agents reduces the amount of circulating PanIN cells and diminishes the number of PanINs in tissue (Tuveson and Neoptolemos, 2012).

The anatomical position of the primary tumor is a crucial determinant in the formation of peritoneal metastasis (Yachida and Iacobuzio-Donahue, 2009; Baretti et al., 2019). In some cases, the exfoliated cells directly attach to and invade organs and tissues in the peritoneal cavity (Jayne, 2007; Yachida and Iacobuzio-Donahue, 2009; Avula et al., 2020). It has been reported that intraperitoneal metastases can also take place via blood vessels or lymphatic absorption through the hematogenous route (Jayne, 2007; Ge et al., 2017). Peritoneal spread of disease is found in around a third of patients with PDAC, which may lead to ascites accumulation in up to 20–30% cases or pleural effusion in around 15% of patients (Golan et al., 2017). Symptomatic retention of fluid with viable cells usually occurs late in the course of the disease, at the clinically treatment resistant phase (Baretti et al., 2019). A detailed analysis of molecular pathways leading to Peritoneal metastasis is reviewed in: (Avula et al., 2020). Briefly, E-cadherin loss, especially in a KRAS mutated background, and HGF/c-Met [hepatocyte growth factor (HGF)/mesenchymal–epithelial transition factor (c-MET)] pathway lead to EMT and cell detachment from the pancreas (Furuyama et al., 2000; Takiguchi et al., 2017; Sato et al., 2020). It is still a challenge to effectively treat peritoneal metastasis, thus further efforts in revealing its mechanism should be addressed in the future. Although there is extensive literature describing ovarian and gastric cancer cell immune evasion through the transition of the peritoneal cavity, this issue has not been exhaustively studied in PDAC.

Several studies prove that the hostile milieu of the liver is particularly preconditioned early to favor the engraftment and growth of disseminated tumor cells (DTCs), so-called pre-metastatic niche (PMN) formation. The formation of PMNs is directed by an intricate series of mutual interactions among the TME and tumor cells, along with the exploitation of recruited and resident cells in secondary target organs. Extracellular vesicles and soluble factors are secreted by the primary tumor or premalignant lesions, even before the initiation of PDAC dissemination. They help to form a supportive niche in the liver by providing vascular docking sites for CTCs enhancing vascular permeability, remodeling the ECM and gathering immunosuppressive inflammatory cells (Houg and Bijlsma, 2018). Hepatic metastases show unique characteristics, such as increased proliferation (Ki67), M2 macrophage infiltration, 3p21.1 loss, downregulation of EMT, and metabolic rewiring (Yang et al., 2021). Reichert et al. (2018) demonstrate, using multiple mouse models, that liver metastases highly depend on P120CTN-mediated stabilization of membranous E-cadherin, while the lung seems permissive to colonization by cells that are not MET-capable. Interestingly, PDAC patients with recurrent lung metastases show significantly better overall survival compared with patients with metastasis at other sites (Yamashita et al., 2015).

Next-generation genome sequencing of untreated pancreatic primary tumors and the corresponding patient metastasis showed that cells triggering distant metastasis are genetically indistinguishable with the different metastatic locus bearing the same driver gene mutations (Makohon-Moore et al., 2017). This implies that post-transcriptional or transcriptional modifications are pivotal to support the intricated series of biological bottlenecks that must be surpassed for PDAC to metastasize (Fidler, 2003; Lambert et al., 2017). DTCs display clonal diversification according to the location of the metastatic foci. Lineage tracing studies revealed that metastases in the lung and liver tend to be monoclonal, while those in the peritoneum and diaphragm exhibit polyclonality due to a different via of dissemination (Kleeff et al., 2016; Knaack et al., 2018). These observations suggest that heterotypic interactions between tumor subclones as well as site-specific selective pressures are both central to influencing metastatic initiation and progression.

Since CTCs do not express β2-integrins, they form clusters with blood cells using them as a linker to attach the capillaries and extravasate to distant organs (Charles Jacob et al., 2021). Extravasation may be controlled by E-selectins, N-cadherin, or galectin-3 from endothelial cells (Yadavalli et al., 2017). Once CTCs extravasate to the secondary organ, they remain dormant with high resistance to current therapies (Sosa et al., 2014). TGF-β and BMPs stromal signals have been identified as promoters of tumor dormancy by enforcing quiescence and inhibiting self-renewal of DTCs (Gao et al., 2012). The perivascular niche has also been reported to induce cancer cell dormancy (Ghajar et al., 2013). In contrast, contexts rich in fibronectin or type 1 collagen inhibit dormancy (Massagué and Obenauf, 2016). A lack of stromal growth factors and an abundance of growth-inhibitory signals can favor metastatic dormancy in experimental models. DTCs are also kept in a dormant state due to constant pressure from the immune system. However, the acquisition of further mutations, inflammation, microenvironmental alterations, as well as immune and stromal signals can promote arouse of dormant cells inducing local relapse or metastases still after curative therapy (Aiello et al., 2017; Lenk et al., 2018; Park and Nam, 2020). Dormany explains why most patients that were resected with no margin experienced a relapse and died of metastatic disease. Thus, it is a future goal to fully understand the pathways leading to metastasis outgrowth and improve current adjuvant combinations to not only eliminate the primary tumor but also to eradicate the dormant foci to prevent relapse.

The formation of metastatic foci occurs with a transition from mesenchymal to epithelial phenotype, leading to enhanced proliferation and metastatic tumor deposit (Makohon-Moore et al., 2017). The aim of adjuvant chemotherapy is the prevention of metastatic relapse. Nonetheless, the current pharmacological armory employed attacks proliferating tumor cells instead of targeting metastasis. Avoiding metastasis in high-risk patients would be ideal rather than treating them. However, the few approved drugs targeting the stroma of metastasis (bisphosphonates, Zometa, anti-RANKL antibody, and denosumab) have not yielded an improvement in the adjuvant setting (Smith et al., 2015; Perrone et al., 2019). Hence, the current standard of care does not instruct any agent to prevent metastasis (Massagué et al., 2017). There are several undergoing clinical trials of targeted agents designed specifically with patients suffering from locally advanced disease or metastasis (Table 1) hoping to improve patients’ overall survival.

### Conclusion

The early dissemination capacity of PDAC, even at the PanIN stage, explains why most patients have already found significant metastasis to the liver, lungs, peritoneum or lymph nodes at the time of diagnosis, and subsequent median survival is less than 1 year (Kleeff et al., 2016; American Cancer Society, 2021). Even though we hold an improved understanding of PDAC biology and progression, the translation for patient benefit has been slow. On this subject, a possible contributing factor may be the lack of solid trustworthy models of human PDAC for preclinical testing and research.

## *In vitro* Preclinical Models

### 2D Cell Lines

Compared to other models, 2D cell culture is the simplest, fastest and most economic form to study metastasis and invasion. There are 49 PDAC cell lines described in Cancer Cell Line Encyclopedia with specific mutations and from different tumor sites (Table 2). Many PDAC cell lines from patients and murine tumors accompanied by different mutations of KRAS, p53, p16, and SMAD4 are widely used in PDAC metastasis research (Sun et al., 2001).

**TABLE 2:** Most common Human derived pancreatic cancer cell lines and metastatic activity in orthotopic transplantation model.

Cell line	Mutation				Sample collection site	Metastasis *in vivo* (orthotopic)	References	Histology	COSMIC ID
	KRAS	TP53	SMAD4	CDKN2A					
PANC-1	G12D	R273H	WT	Hom. Del.	Pancreas	Liver, lungs	Loukopoulos et al., 2004; Zhang et al., 2020	Ductal carcinoma	1366282
AsPC-1	G12D	C135Afs*35	R100T	L78Hfs*41	Ascites	Lung, Liver, Lymph nodes	Loukopoulos et al., 2004	Ductal carcinoma	910702
BxPC-3	WT	Y220C	Hom. Del.	Hom. Del.	Pancreas	Lung, Liver, Lymph nodes	Loukopoulos et al., 2004; Razidlo et al., 2015	Ductal carcinoma	906693
CAPAN-1	G12V	A159V	S343*	Hom. Del.	Liver	Lung Liver Lymph nodes	Loukopoulos et al., 2004	Ductal carcinoma	753624
CAPAN-2	G12V/-	T125	WT	T18A19dup	Pancreas	Liver	Loukopoulos et al., 2004	Ductal carcinoma	910915
CFPAC-1	G12V/-	C242R	Hom. Del.	–; promoter methylation	Liver	Lung, Liver, Lymph nodes	Loukopoulos et al., 2004	Ductal carcinoma	906821
HPAC	G12D	G187R	D52Rfs*2	p.E120*	Pancreas	Lung, liver, peritoneum	Kuo et al., 2021	Carcinoma	1298136
HPAF-II	G12D/-	P151S	WT	R29A34del	Ascites	Lung, Liver, Lymph nodes	Fujisawa et al., 2009; Massey et al., 2019	Ductal carcinoma	724869
Hs766T	Q61H	WT	Hom. Del.	WT	Lymph node	Lymph, Liver, peritoneum, ascites	Fujisawa et al., 2009	Carcinoma	1298141
MIA-PaCa-2	G12C	R248W	WT	Hom. Del.	Pancreas	Lung, Liver, Lymph nodes, Peritoneum	Higuchi et al., 2017	Ductal carcinoma	724870
MZ1-PC	G12V	R209Kfs*6	–	R80*	Pleural Effusion	–	–	Ductal carcinoma	753595
PANC-02–03	G12D/-	R248Q	R135*	Y44*	Pancreas	–	–	Carcinoma	1298475
PANC-03–27	G12V/-	c.375 + 5G > T	–	Hom.Del.	Pancreas	–	–	Ductal carcinoma	925346
PANC-04–03	G12D/-	G245S	–	Y44*	Pancreas	–	–	Carcinoma	1298476
PANC-08–13	G12D	–	C123Mfs*2	–	Pancreas	–	–	Ductal carcinoma	925347
PANC-10–05	G12D/-	I255N/-	–	–	Pancreas	–	–	Ductal carcinoma	925348
PA-TU-8902	G12V/-	C176S	–	–	Pancreas	–	–	Carcinoma	1298526
PA-TU-8988T	G12V	R282W	Hom.Del.	–	Liver	Liver, kidney (SC)	Miao et al., 2020	Carcinoma	1240201
PL18	WT	E171del, R267W	–	–	Pancreas	–	–	Pancreatic adenocarcinoma	1240208
PL4	G12D	G266V	–	–	Pancreas	–	–	Carcinoma	1298533
PSN1	G12R	K132Q	Hom.Del.	Hom.Del.	Pancreas	Liver	Eyres et al., 2021	Ductal carcinoma	910546
SU8686	G12D	G245S, p.G360V	–	Hom.Del.	Liver	Primary	Liu et al., 2020	Carcinoma	1240218
SUIT-2	G12D	R273H	–	E69*	Liver	Liver, Lung, Kidney, Peritoneum	Higuchi et al., 2017	Carcinoma	1240219
SW1990	G12D	P191del	–	Hom. Del.	Spleen	–	–	Ductal carcinoma	910907
YAPC	G12V/-	H179R	R515Dfs*2	–	Ascites	–	–	Ductal carcinoma	909904

*Hom.Del. homozygous deletion, ^∗^ deletion, WT Wild Type, and – unknown.*

Several migration and invasion assays can be performed in cell lines to study metastasis. A fast method to assess migration in 2D cell culture is the wound healing assay, where a scratch is performed in the middle of confluent monolayer cells, and the measurement of cell migration is quantified via microscopy. Using this method several groups were able to identify genes that promote motility namely Yes1 associated transcriptional regulator (YAP1), H19 imprinted maternally expressed transcript (H19) or C-X-C motif chemokine ligand 12 (CXCL12; Cecati et al., 2021; Gao et al., 2021; Luo et al., 2021). However, this method is not able for non-adherent cells and the scratch can cause cell damage. Alternatively, invasion can also be studied using inserts in which cells are seeded on top and invasive cells are able to pass through such as the Transwell or Boyden chamber. To reproduce ECM degradation, it is possible to add a layer of ECM in the inserts (Kramer et al., 2013). Several studies analyzed the invasive capacities of PDAC cell lines through several substrates. Quite unanimously, Capan-1 and Mia Paca-2 have the higher invasion rates followed by PANC-1 and BxPC-3 with slight variations between groups due to subtle differences in methodology (Deer et al., 2010). It is also possible to study the morphology, directionality and velocity of migrating cells using optical mobility assays such as the TAXIScan (Yamauchi et al., 2017). Using this method, it has been shown that only a few cells are able to invade the ECM but they up-regulate the expression of invasion-promoting pathways such as PI3K-AKT (Fujita et al., 2014).

Another benefit of 2D cultures is the capacity to co-culture cancer cells with stromal cells, and model the signals from the TME. For example, co-culturing cancer cells with Pancreatic stellate cells (PSCs) showed that cancer cells had enhanced EMT markers and migration (Kikuta et al., 2010). Recently, the development of microfluidic assays has allowed investigating the biophysical parameters in PDAC metastasis modeling the chemical gradients, flow/shear stress and the complex interactions between several cell types (Kramer et al., 2019). Using PDAC cell lines and combining the previously mentioned assays with CRISPR-Cas9 technique has revolutionized the field of metastasis research. Currently, there are available genome-wide or custom made sgRNA CRISPR libraries that have helped identify genes promoting migration, chemoresistance or radioresistance such as Histone Deacetylase 1, ATP binding cassette subfamily G member 2, Endoplasmic reticulum-associated protein degradation (Du et al., 2021; Ramaker et al., 2021).

Lack of germline DNA and missing clinical annotation are general problems when working with established cell lines. Since 2D cell lines are separated from tissue and cultured on a flat cell culture surface, they undergo several *in vitro* selection steps. They will divide abnormally, become flat, and lose their differentiated phenotype (Kapałczyńska et al., 2016). Thus, some cell types are not well-represented and the tumor heterogeneity is reduced. Recently, single-cell sequencing technologies showed that there is a high degree of heterogeneity in commonly used PDAC cell lines, induced by culturing identical PDAC cell lines in different laboratories. They also question the use of immortalized, non-transformed pancreatic lines as control lines in the experiments (Monberg et al., 2021). Despite their limitations, cell lines have been pivotal tools for screening in pre-clinical settings the genes promoting migration and survival in PDAC as well as chemo- or radioresistance.

### Spheroids

Despite being time and cost-effective, 2D cell cultures do not represent the architecture and structural complexity of human tissues. 3D culture of normal cells and their neoplastic counterparts was introduced in the 1970s (Emerman and Pitelka, 1977). Since the development of the hanging drop technique, spheroids have been utilized to study morphogenesis together with the composition and architecture of malignant tissues (Kelm et al., 2003) using several techniques summarized in Table 3. In semisolid matrices resembling the basement membrane, cell-cell contacts and cell-matrix interactions allow epithelial cells to develop polarized structures. Abounding ECM components namely collagen, fibronectin and laminin are necessary to support these cultures. Interestingly, studies that have compared transcriptomes between 2D and 3D cultures have shown that cells are highly influenced by cell-matrix interactions. Several PDAC cell lines are able to grow in spheroids but it is unclear their ability to reflect the properties of the human tumor since they undergo deep transcriptomic transformations in transitioning from 2D to 3D (Monberg et al., 2021).

**TABLE 3:** Three-dimensional spheroid models for PDAC research and their applications in metastasis.

	Technique	Application	References
PDAC Spheroids model	Modified Hanging drop	PDAC-stroma interaction analysis and HT automated drug screening assays	Ware et al., 2016
	PANC-1 co-culture with mPSCs	Test chemosensitivity to gemcitabine, paclitaxel, and SN38	Liu et al., 2021
	Co-culture (type I collagen)	Stroma-mediated cell motility and drug resistance	Lee et al., 2018
	Co-culture (CS-HA coated plates)	Cellular interaction, migration, and drug resistance	Wong et al., 2019
	Co-culturing with microtissues	A testing platform for anticancer drugs in Tissue-on-chip technology	Brancato et al., 2017
	User-defined tumor compartment embedded in 3D matrix	A High-throughput testing platform for anticancer drugs screening	Puls et al., 2018

Pancreatic stellate cells are the main source of stromal fibrosis, interacting closely with cancer cells to produce a supportive environment that drives local and remote neoplastic development. By co-culturing PDAC spheroids with ECM components, it is possible to model PDAC-stroma interaction. Some groups have shown that PSC co-cultured spheroids reflect PDAC chemotherapeutic responses (Lee et al., 2018; Wong et al., 2019; Liu et al., 2021). For instance, PSC/PDAC spheroids showed enhanced resistance to gemcitabine in comparison to PDAC-only spheroids, while c-MET inhibitors crizotinib, tivantinib, and PHA-665752 were similarly effective in both models (Firuzi et al., 2019). Recently, other groups also proved the increased chemosensitivity from heterospecies spheroids to gemcitabine, paclitaxel and SN38. Interestingly, this group also showed that upon co-culture mPSC induces a shift from classical to a basal-like phenotype to PANC-1 spheroids showing the importance of TME in patient prognostic and metastasis development (Liu et al., 2021). CAFs are activated to myofibroblast and tumor-dependent lymphocyte infiltration is observed on co-culture reproducing the desmoplastic reaction of PDAC and providing a valuable tool for anticancer drug testing (Brancato et al., 2017; Tsai et al., 2018).

Immune cells interfere in treatment response and tumor progression (Qiu and Su, 2013). 3D approaches admit the inclusion of human immune cells in contrast to patient-derived xenografts (PDXs) which are established in immunodeficient mice. When T cells were added to monocyte and fibroblasts co-cultured with PDAC spheroids overexpressing Doublecortin-like kinase 1 -isoform 2, M2 monocytes were polarized via cytokine release which then inhibits CD4+ and CD8 + T cell activation and proliferation (Kuen et al., 2017; Chandrakesan et al., 2020). Since knockdown of DCLK-isoform2 resulted in enhanced CD8 + T cell activation and decreased pancreatic cancer cell viability—this study with spheroids suggested DCLK-isoform2 as a novel therapeutic target in PDAC (Chandrakesan et al., 2020). Importantly, another triple co-culture platform has been developed combining PANC-1, endothelial cells (HUVEC) and fibroblasts (MRC-5) which also mimicked the resistance to treatments observed *in vivo* to doxorubicin and gemcitabine hence proving the key role of a complex tumor microenvironment (Lazzari et al., 2018).

With additional therapies for stroma targeting and 3D patient models that replicate a patient’s specific TMEs, it is an exciting time for PDAC research. Several Clinical Trials target the tumor microenvironment of PDAC (Tomás-Bort et al., 2020). 3D cell cultures, and specially PSC/PDAC spheroids, are important tools for screening of cancer and stroma targeting drugs permitting a validation step preceding animal testing and reduce the number of animals required (Ishiguro et al., 2018). 3D modeling of cell culture may aid in drug discovery and biological treatment. While current 3D spheroid invasion models more precisely replicate tumor invasion compared to conventional 2D models, they have limitations such as low reproducibility and the difficulty to interact with high-throughput (HT) systems.

To overcome this limitation, Puls et al. developed a 3D tumor-tissue invasion model for HT phenotypic drug screening. In short, PDAC cell lines are embedded in an Oligomer in suitable plates for HTS where it is possible to monitor invasion into the surrounding tissue. When CAFs were added this highly enhanced PDAC invasion, as is expected to occur *in vivo*. Additionally, they showed that gemcitabine inhibited proliferation while not fully eradicating the tumor or blocking invasion. These results line up with those from PDAC xenograft models which show gemcitabine substantially arrests tumor growth and proliferation but does not induce apoptosis or reduction of remote metastases and invasion related markers (Puls et al., 2018). Although 3D spheroids have proven useful in cancer cell research, it is acknowledged that a passive environment does not adequately represent the cellular development of these cancer cells. The tumor cells grew throughout time without being suppressed by the drugs; however, it is not clear how much of the development was hindered or accelerated as a result of static media supply (Holub et al., 2020).

### Organoids

Inferring results from model systems to humans has been a major barrier in the drug discovery process. In the last decade, a surrogate *in vitro* 3D model for human and mice tissues, named organoids, has been refined. Unlike spheroids, organoids are not derived from cell lines but from primary cells. Moreover, organoids allow studies of tissue function since tissue-like structures are preserved (Marsee et al., 2021). Stem cells are isolated from mouse or human adult tissues and embedded in 3D matrices where they self-organize into epithelial structures (Tuveson and Clevers, 2019; Kim et al., 2020). They also maintain intra-tumor heterogeneity, cell polarity and interact with the ECM, resembling the molecular features of the original tumor. Not long ago, organoid cultures of pancreatic epithelium have allowed the culture of normal and neoplastic pancreatic epithelial cells for both humans and mice (Huch et al., 2013; Hindley et al., 2016; Boj et al., 2018).

To establish organoid cultures, it is necessary to mimic the homoeostatic surrounding of the normal tissue stem cells. For this purpose, cells are encapsulated in Matrigel, which contains the crucial components of the basement membrane, and complemented with the minimal essentials for sustainable growth of pancreatic epithelial cells left out mesenchyme. Since the majority of PDAC samples have high penetrance of KRAS activation (Yachida and Iacobuzio-Donahue, 2009), it is possible to apply selective pressure conditions withdrawing EGF or adding EGFR inhibitors to obtain a pure neoplastic culture.

Since organoids are genuine epithelial populations, they bypass the stromal suppression that primary tumors retain, allowing comparisons to normal ductal pancreatic cells (Boj et al., 2018). They can be established in several weeks even from small fine needle aspiration biopsies acquired from patients with advanced PDAC, enabling therapeutic testing and tumor response during treatment or disease progression. Employing a large cohort of PDOs, Tiriac et al. (2018) have developed a platform for testing single and targeted agents. They display, in retrospective case studies, that organoid response to therapeutic testing correlates with patient sensitivity to chemotherapy. By correlating the transcriptome and drug sensitivity profile of each organoid in the cohort, they defined transcriptomic signatures of chemosensitivity with prognostic clinical outcomes in treated cohorts of PDAC patients. Other authors found the same concordance when testing similar platforms (Huang et al., 2015; Romero-Calvo et al., 2019; Dreyer et al., 2021b). In PDOs obtained over multiple years in a metastatic PDAC patient, it was possible to show increased organoid resistance to chemotherapy in accord with treatment refractory cases (Tiriac et al., 2018). Organoid work has also shown that Beta-1,4-galactosyltransferase 1 (B4GALT1) promotes PDAC progression and chemoresistance via stabilization of CDK11^*p*110^ (Chen et al., 2021, 110). In the biomarker field, organoids showed that higher EV release is coupled to a high cell proliferation rate, promoted by Wnt pathway activation (Sándor et al., 2021).

Since their implementation, organoids have been able to demonstrate good genomic parallelization with the primary PDAC tumors (Tiriac et al., 2018; Gendoo et al., 2019; Romero-Calvo et al., 2019). Also, PDAC subtypes have been identified in independent cohorts of PDOs implying that the transcriptional programs are preserved. Seino et al. (2018) defined functional subtypes of PDAC and demonstrated an inverse correlation between and strict requirement for WNT-signaling and GATA6 expression (linked with classical subtype), thus implying that GATA6 acts as a key regulator of niche-dependency. This emphasizes the call for precision methods to select patients when considering Wnt pathway therapeutic approaches, for example with the porcupine clinical trials. In addition, organoids are genetically manageable for viral infection and transfection, allowing targeted evaluations of particular genes or genetic screening (Michels et al., 2020).

Co-cultures of organoids with PDAC stromal cells helped understand fibroblast heterogeneity and suggested new approaches for treating PDAC by blocking the fibroblasts that support the tumor and promoting tumor restraining fibroblasts (Öhlund et al., 2017; Tsai et al., 2018; Biffi et al., 2019). These co-culture conditions have also shown that CAFs modify the EMT phenotype and drive gemcitabine resistance induced by HGF derived from CAFs. Furthermore, high stromal expression of Paired related homeobox 1 (Prrx1), a transcription factor critical for activating CAFs, is displayed in the squamous subtype (Feldmann et al., 2021). Using organoids and mice, Walter et al. showed that MEK inhibition suppresses TGFβ-induced EMT and migration *in vitro* and eventually results in a greater decrease in CTCs *in vivo* (Walter et al., 2019). Further studies in PDAC metastasis have been achieved by creating organoid derived xenografts (ODX; section “*Organoid derived xenografts”*).

The recent findings prove that organoids should be a focal point of future studies of PDAC. Overall, organoids recapitulate the human disease much closer than spheroids or cell lines. They allow tissue function studies and co-culture. Since it is possible to culture the normal and neoplastic compartment, organoids are well suited for therapeutic testing and have intermediate scalability. However, organoids are still a complex model that requires a lot of technical training and represent a high cost to establish and maintain (Marsee et al., 2021). Despite showing correlation with patient transcriptomics subtypes and chemoresistance signatures, it has been shown that there are transcriptomic switches during *ex vivo* passage that may restrict their predictive abilities (Monberg et al., 2021), thus correct passage monitoring is required. To bring to the clinic fast organoid testing of PDAC patients, further work is required in accelerating organoid establishment and testing techniques of valuable compounds.

Recently, a consortium named PRECODE (European Commission, 2021) was established where several laboratories collaborate working on Pancreatic Cancer Research in Organoids in different fields helping to push forward the understanding of this disease and get closer to the development of an effective treatment for PDAC.

## *In vivo* Preclinical Models

*In vivo* models are key to study alternative and innovative treatment approaches. Despite the great advantages of *in vitro* research, namely cost-efficiency and simplicity, these models are lacking a microenvironment, immune system and don’t represent tumor heterogeneity. Thus, *in vivo* models have been widely used to overcome these limitations for metastasis research and allow the understanding of the complex crosstalks involved in metastasis and defining its stages.

### Genetically Engineered Mouse Models

Transgenic models, using tissue or cell-type specific promoters, allow the ectopic and temporal expression of target genes in the mouse genome. Different pancreatic cancer lineage specific promoters have been employed in GEMMs like pancreatic and duodenal homeobox 1 (Pdx1), neurog3 (Ngn3), elastase (Ela), among others.

While several chemical and genetic approaches to generate PDAC in mice date back to the 1980s (Longnecker, 1984), it was the establishment of the Kras^*LSL.G*12*D*^ mice (Johnson et al., 2001) in 2001 that permitted tissue-specific expression of oncogenic Kras under physiological control from the endogenous mouse locus. This model developed Lung cancer but not PDAC. After this, several GEMMs faithfully recapitulating the genetic, molecular, histological, and clinical hallmarks of human PDAC have been established (Table 4). A full review of GEMMs for PDAC is available in: (Westphalen and Olive, 2012).

**TABLE 4:** Genetically engineered mouse models of pancreatic cancer summary of the most common GEMMs of PDAC driven by KrasG12D.

Name	Mutation	Phenotype	References
KC model (Kras^*LSL.G*12D/+^; PdxCre)	Oncogenic Kras	Pre-invasive PanIN to PDAC	Hingorani et al., 2003
Kras^*LSL.G*12D/+^; Cdkn2a^*lox/lox*^; PdxCre	Oncogenic Kras, homozygous or heterozygous deletion of Cdkn2a	Rapid metastatic PDAC	Aguirre, 2003
KPC model (Kras^*LSL.G*12D/+^; p53^*R172H/+*^; PdxCre)	Oncogenic Kras, heterozygous deletion of Trp53	Pre-invasive PanIN to metastatic PDAC	Hingorani et al., 2005
(Kras^*LSL.G12D/+*^; Ink4a/Arf^*lox/+*^; PdxCre)	Oncogenic Kras, heterozygous deletion of Ink4a/Arf	Rapid metastatic PDAC	Bardeesy et al., 2006b
(Kras^*LSL.G*12D/+^; Tgfβr2^*flox*^; PdxCre)	Oncogenic Kras, homozygous deletion of Tgfbr2	PDAC with liver metastasis	Ijichi et al., 2006
KD model (Kras^*LSL.G*12D/+^;Dpc4^*flox/+*^;p48Cre/+)	Oncogenic Kras, heterozygous deletion of Smad4/Dpc4	MCNs to metastatic PC	Izeradjene et al., 2007
KPCZ model (KPC; Zeb1^*fl/fl*^)	Oncogenic Kras, heterozygous deletion of Trp53, and homozygous deletion of Zeb1	Decreased local invasion and reduced metastatic competence	Krebs et al., 2017

While transgenic mice are fast to develop and permit the expression of human genes (Qiu and Su, 2013), the expression occurs under foreign promoters. To circumvent these limitations, conditional models are used expressing the desired mutations within the endogenous locus by interbreeding mice carrying the mutant allele downstream of a “Lox-STOP-Lox” (LSL) cassette with Cre driver mice (Magnuson and Osipovich, 2013). In 2003 (Hingorani et al., 2003) by crossing PdxCre or p48Cre mice to Kras^*LSL.G*12*D*^ mice the expression of Kras^*G*12*D*^ was specifically targeted to the pancreas. In the Kras^*LSL.G*12*D/+*^; PdxCre model (KC model), mice are born with normal pancreas but develop PanIN at 8 weeks and slowly increase in grade, with a subset of those developing PDAC. The KC model proved that Kras mutations are enough to initiate PDAC formation in mice while targeted conditional mutations in Cdkn2A, Smad4, or p53 did not lead to PanIN or tumor development with PdxCre expression. However, this long latency, background tumors and sporadic progression to metastasis hampered the utility of the KC model for preclinical applications.

By combining oncogene activation and tumor suppressor inactivation, it has been successful to generate metastatic PDAC models resembling human disease. Aguirre (2003) showed that homozygous deletion of Cdkn2a in the context of Kras mutation in the pancreas (Kras^*LSL.G*12*D/+*^; Cdkn2a^*lox/lox*^; and PdxCre) led to the rapid development of metastatic PDAC. Similarly, the loss of the Ink4a/ARF locus in Kras mutant mice promotes NF-kB, Notch signaling and metastasis (Bardeesy et al., 2006a). Conditional expression of p53^*R*172*H*^ also accelerated Kras^*G*12*D*^ pancreatic tumorigenesis. Although Kras^*LSL.G*12*D/+*^; p53^*R*172*H/+*^; and PdxCre mice (KPC mice) are born with histologically normal pancreas, they rapidly develop PanIN lesions, and die of PDAC in 5.5 months with ∼80% metastasis (Hingorani et al., 2005). This model showed indices of widespread genomic alterations, a characteristic that was previously missing in most GEMMs. Since KPC mice mirror the dynamics of the human TME, they are useful to study tumor-stroma interactions as well as disease progression and testing immunotherapies. Other models addressed the deletion of Smad4 in Kras mutant pancreatic cells but the histology of those tumors was more similar to MCNs or IPMNs (Bardeesy et al., 2006b; Izeradjene et al., 2007). Interestingly, homozygous deletion of transforming growth factor beta receptor 2 (Tgfbr2) combined with Kras^*G*12*D*^ formed PDAC with metastasis with special tropism to the liver, suggesting that activated Ras signaling and hampered TGF-β signaling cooperate to advance PDAC progression (Ijichi et al., 2006).

Classical Cre-loxP GEMMs depend on a single Cre-mediated step of recombination to activate oncogenic Kras expression not allowing sequential multistep tumorigenesis and tumor heterogeneity, which are important hallmarks of PDAC. With a dual-recombinase system (Schönhuber et al., 2014), Krebs et al. (2017) generated the KPC; Zeb1^*fl/fl*^ mice (termed KPCZ) model and discovered that the EMT-TF Zinc Finger E-Box Binding Homeobox 1 (Zeb1) is a crucial factor for driving metastasis.

Genetically engineered mouse models have enlightened the biology of PDAC, elucidated potential therapeutic and diagnostic targets, and accentuated the importance of the tumor stroma for pancreatic cancer immune evasion, maintenance, and drug resistance. Nonetheless, it is an expensive and labor-intensive model to generate and maintain. In addition, gene mutations are brought into the germline of mice, while they occur somatically and gradually in human tumors. Nonetheless, these limitations may be overcome by the use of CRISPR-Cas9 in mouse models. Recently, CRISPR-Cas9 technology has allowed more precise genome editing (Doudna and Charpentier, 2014; Platt et al., 2014). Using this method, Ideno et al. developed the Ptf1-Cre; LSL-Cas9 mouse model, which recapitulates human PDAC features such as PanIN or IPMN with potential advancement to PDAC (Ideno et al., 2019). Ischenko et al. (2021) used CRISPR-Cas9 to inactivate Kras in mice and demonstrated that in advanced tumors, Kras tumor growth dependence is diminished and is shown in the suppression of antitumor immunity.

All these models prove the crucial role of KRAS in the biology of pancreatic cancer; even though efforts to target KRAS directly have not been successful to date. Thus, Ras effector pathways namely Phosphatidylinositol 3-kinase (PI3K)- Protein Kinase B (AKT) and Raf- Mitogen-activated protein kinase kinase (MEK)- Extracellular signal-regulated kinase (ERK) have been investigated as potential surrogates (Mann et al., 2016). Following this line, by crossing to KC mice and analyzing transposon insertions in the resulting tumors, it has been described a large number of candidate genes that may promote tumor progression in Kras^*G*12*D*^ initiated pancreatic tumors namely TGF-β and p16/CDKN2A. Genes implicated in chromatin remodeling were identified, including Ubiquitin Specific Peptidase 9 X-Linked (Usp9X), which plays an important role in the pathogenesis of PDAC (Pérez-Mancera et al., 2012; Mann et al., 2012). Several groups followed Usp9X and showed its association with worse prognosis in PDAC (Liu et al., 2017b; Pal et al., 2018).

The grade of aneuploidy in human tumors leads to a great variety of intertumoral gene modifications, with a completely different appearance as in mice. Overall, these species-related differences hamper the capacity of GEMMs to predict the true therapeutic response of PDAC patients in clinical trials. To overcome these limitations, transplantation models might be used.

### Transplantation Models

Transplantation models consist of the engraftment of mouse or human cells/spheroids/organoids/tissues into recipient mice. This provides the benefit of tractability and a relatively lower and more predictable tumor latency than transgenic models. The transplantation can be orthotopic (in the pancreas), or heterotopic (subcutaneous, intraperitoneal, intravenous, intrasplenic, or intra-cardiac) according to where the cells are implanted. Cells engrafted via orthotopic transplantation may spread from the primary tumor to remote organ sites, hence permitting the modeling of the entire metastatic cascade; whereas when injected heterotopically into circulation it is possible to reproduce the steps of dissemination, extravasation, and colonization (Gómez-Cuadrado et al., 2017). Different sites of vascular injection define the site of colonization. For example, injection of cancer cells in the tail vein leads to the development of lung metastases since the cells are rapidly trapped in the microvasculature of the lung. Intrasplenic injection leads to the formation of micrometastasis in the liver. On the other hand, intracardiac injections allow systemic dissemination and are used to model brain or bone metastasis (Khanna, 2004). Moreover, transplantation models can be xenogeneic (xenograft) or syngeneic (allograft).

Allograft transplantation models are established by the transplantation of mice derived neoplastic cells and tumors into mice. They permit the study of metastatic dissemination with an intact immune system, and hence more closely recapitulate the TME. Allografts from isolated cancer cells or tumor pieces derived from GEMMs were characterized by a faster and more consistent development of primary tumors and up to 90% metastasis in the liver compared to GEMMs (Li et al., 2019). The abundance of metastasis in this model is probably a result of focal disease formation, closely mimicking the random mutations in KRAS present in human disease (Tseng et al., 2010).

In contrast to allograft models, xenografts require the implantation of human tumors or cancer cells into immunocompromised mice. Pancreatic cancer cell lines or spheroids are a frequent source for transplant. Nonetheless, as phenotypic and molecular properties may switch in culture, xenograft models of cancer cell lines do not always anticipate clinical responses (Garcia et al., 2020) and thus 3D models are a better alternative.

### Cell Line Derived Xenografts and Spheroid-Based Xenografts

One of the solutions to address the many shortcomings of 2D cell lines is to establish cell line derived xenografts (CDX). PDAC cell lines are implanted into mouse models to research and test the efficacy of anti-cancer therapies *in vivo* and metastasis formation. Several studies produced allograft models with C57BL/6, such as TB 32047 (Lu et al., 2020), KPC cell line (Torres et al., 2013), or Pan02 (Jiang et al., 2014). In contrast to allograft models, human PDAC CDX are established by transplanting PDAC cell lines into immunocompromised mice. Resuspended cells in Matrigel for injection to establish an orthotopic mouse model of PDAC is a common method. The orthotopic injection of SUIT-2 cells into the pancreas can induce a process similar to the spread of human PDAC (Higuchi et al., 2017). 3–14 days after inoculation, Higuchi et al. observed intraperitoneal dissemination, extrapancreatic invasion, and further hematogenous organ metastases of SUIT-2 cells (Table 1).

The lung and liver are the most common sites of metastatic PDAC at diagnosis. Most CDX models are generated by subcutaneously injecting PDAC cells into immunodeficient mice. However, subcutaneous xenograft tumors rarely metastasize and thus orthotopic models are a better alternative (Table 1). PANC-1 and KP3, AsPC-1 and KP2 develop liver metastases while only AsPC-1 showed signs of lung metastases (Zhang and Du, 2019). Interestingly, multinucleated cells and spindle cells have been observed in liver and lung metastases playing an important part in metastasis formation. There is also a lung metastasis model by injecting PDAC cells via the tail vein (Kong et al., 2020). Metastatic tumors can be observed in the lungs and other organs after about a month.

Pancreatic ductal adenocarcinoma orthotopic metastasis mouse models are successfully established by injection 2D cells into pancreas. There have also been some studies that xenografted 3D spheroids from PDAC cells (Durymanov et al., 2019; Azmi et al., 2020). 3D spherical culture, as opposed to classic monolayer cell culture, more nearly replicate *in vivo* conditions inside a microenvironment, which can improve the defects of 2D culture (Liu et al., 2021). Furthermore, in contrast to their cell-based counterparts, spheroid-based xenografts (SDX) show increased expression of pro-fibrotic and pro-survival PDAC hallmarkers (Durymanov et al., 2019). Orthotopic implantation can progress pancreatic tumor to liver and lung metastasis tumor, which is similar to humans. But some PDAC cell lines are difficult to metastasize. Tail vein and splenic injection can easily perform metastasis, but it doesn’t produce a primary tumor. Secondly, immunodeficient mice successfully avoided rejection during xenotransplantation, but it also limits the study of metastasis progress since adaptation to the immune system plays an important role in the selection of metastatic mutations (Gonzalez et al., 2018). Another drawback of the CDX-SDX models is that they may not achieve the medical requirements of individualization and precision. Cell lines cannot accurately reflect the complexity of tumor heterogeneity and can only represent patients with certain types of cancer (Xu et al., 2018).

### Organoid Derived Xenografts

Orthotopic transplantation of human pancreatic tumor organoids into immunocompromised mice mimics the full spectrum PDAC progression, forming PanIN-like stages and advancing to invasive and metastatic PDAC (Boj et al., 2018). The only difference is that PanIN-like structures are not intraductal epithelial structures within the pancreas of the mouse. Conventional CDXs only repopulate the host environment but do not form any PanIN-like structure. In addition, ODXs closely recapitulate the dense collagen deposition present in human PDAC tissues and tumors from GEMMs, another feature missing in CDXs (Kim et al., 2009; Olive et al., 2009; Raimondi et al., 2020).

It is still unknown how engrafted PDAC organoids form PanIN-like structures. As organoid cultures can better retain tumor cell heterogeneity, it is possible that recovering several stem cells better mimics the different stages of disease progression upon implantation. In addition, it could be the organoid culture conditions that better reflect the cellular plasticity and epigenetic changes upon transplantation. The interaction of the matrix with pancreatic cancer cells in organoid cultures may help to switch into a PanIN-like biological stage (Hwang et al., 2016). Differentially from other xenograft models, ODXs offer a unique chance to study PDAC progression *in vivo* and early biomarkers. Undergoing research will evaluate the benefits of the ODXs for therapeutic and diagnostic development in comparison to classical PDAC models.

### Patient-Derived Xenografts

Patient-Derived Xenografts have been established as a rising tool to recapitulate tumor heterogeneity, genetics, and cancer microenvironment of PDAC. PDXs are used to identify new biomarkers, enhance therapeutic outcomes and also as tools for personalized treatments of PDAC patients.

Patient-derived xenografts from patient tumor tissue represent a more favorable alternative to CDX, SDX, or ODX since there is no *in vitro* selection. Patient tumor pieces are implanted into immunocompromised mice orthotopically or subcutaneously for propagation *in vivo*, followed by passage of tumor fragments in subsequent generations (Tentler et al., 2012). PDAC PDXs develop between one and 4 months after transplantation, with an engraftment rate between 20 and 80% (Garcia et al., 2020). They have been shown to conserve metastatic potential and histology of the original tumor (Hidalgo et al., 2014) and closely mirror drug responses in human patients (Voskoglou-Nomikos et al., 2003). This may represent the fact that PDXs are not composed of cancer cell populations separated from human tumors and adapted to culture conditions. Since PDX models are established from tumor fragments, the tumor neoplastic cell architecture is retained (Loukopoulos et al., 2004; Rubio-Viqueira and Hidalgo, 2009). Although the initial human stroma is gradually replaced with cells of the murine host, these models can recapitulate the complexity of the TME in PDAC (Duda et al., 2004). Also, successively passaged PDXs normally show consistent biological properties and homogeneous histological and molecular phenotypes (Aparicio et al., 2015). Since it is necessary to use immunocompromised mice when generating xenografts, this represents a major drawback to study metastasis, since the adaptive immune system plays a crucial role in the selection of metastatic variants (Gonzalez et al., 2018).

### Chick Chorioallantoic Membrane Xenografts

Chick chorioallantoic membrane (CAM) is a deeply vascularized extraembryonic membrane formed after embryonic day 5 rich in type IV collagen and laminin, which are similar to the human basement membrane. Chick embryos are not immune-competent until day 18 (Ribatti, 2016). More and more studies have confirmed that the CAM model can efficiently sustain tumor cell proliferation, making it a simple and rapid model for studying initial tumor development. It can reproduce all stages of tumor formation in a shorter amount of time as tumors can be detected after only 4 days of cancer cell injection (Komatsu et al., 2019). It has been shown that firefly luciferase-labeled primary PDAC cells can be engrafted onto the CAM with >80% success. A comparison of tumors harvested from the CAM with original human tissues by immunohistochemical staining showed similar positive staining for the PDAC markers cytokeratin, Cytokeratin 19, Cytokeratin 7, mucin-1, and Alcian blue. Importantly, the percentages of positive/negative cells within each model are very consistent (Rovithi et al., 2017). In addition to using fluorescently labeled cells, cell invasion can be analyzed by Alu PCR to estimate the presence of metastatic human cancer cells in the organs of the chick embryo. Li et al. extracted genomic DNA from chick CAM and liver, respectively, and Alu sequences in human cells were specifically detected by Alu PCR revealing that 31% of CAM and 0% of liver tissue were Alu positive in embryos with untreated Aspc-1 cells. After dexamethasone treatment, the invasion rate of Aspc-1 tumor cells to CAM and liver increased metastasis to 85% and 60%, respectively (Mira et al., 2002; Liu et al., 2017a).

In summary, CAM-assay is a flexible, cost-efficient, reproducible and rapid approach that can evaluate the metastatic capacity and aggressiveness of different PDAC cells within a short time *in vivo*. With the help of physiological and histological characteristics of PDAC, it is easy to assess the key features of tumor metastasis such as angiogenesis, intravasation and spontaneous metastasis. Therefore, CAM assay is an attractive metastasis model. Immune deficiency of chick embryos is up to 18 days, so the short observation periods (3–9 days) become an important limitation of CAM-assay. In addition, it cannot examine cancer-immune cell interactions. Another limitation to this system is that chick embryos are extensively vascularized organisms characterized by fast morphological changes (Lokman et al., 2020). CAM-assay for tumor research is also limited in its monitoring capabilities of tumor size. Because the tumor is encased by a radiopaque eggshell and has a modest structural size, it can only be monitored from above, posing a challenge to existing imaging modalities. Even though repetitive ultrasonography can monitor tumor growth and vascularization, it also relies on the experience of the experimenter in ultrasound (Eckrich et al., 2020).

### ZebraFish Model

Recently, the value of the zebrafish model has been appreciated. *Teleost zebrafish* (Danio rerio) shows large levels of physiologic and genetic analogies to mammals, closely resembling the clinical setting and allowing the natural history of the tumor to be monitored (Wu et al., 2017). Fishes are routinely maintained at 28°C, but the most favorable temperature for tumor cell proliferation is 37°C (Cabezas-Sainz et al., 2018). When engrafted zebrafish are raised at a compromise temperature (≤34°C), cancer cells do not proliferate at the same rate as when cultured in mice or humans. The Protein Kinase, DNA-Activated, Catalytic Subunit (prkdc) and Interleukin 2 receptor (il2rga) deficient zebrafish model can be raised at 37°C and can engraft a wide range of human malignancies (Yan et al., 2019). CRE/LOX technology and GAL4/UAS systems were combined to create the first kras-initiated PDAC model in zebrafish that highly recapitulates human PanIN development (Oh and Park, 2019). A xenograft model was established in zebrafish by transplanting human pancreatic cancer cells into the perivitelline cavity of 48 h post-fertilization zebrafish embryos (Guo et al., 2015). Subsequently, they observed that cells with kras mutations displayed significant proliferative and migratory behaviors invading the zebrafish vasculature system. Then xenotransplanted larvae were exposed to an inhibitor that targets the KRAS signaling pathway named U0126. There were fewer metastases in the bodies of the larvae in the following U0126 treatment group while the mock-treatment group displayed recurrent metastasis.

Zebrafish is a useful and economical *in vivo* animal model for speedy analysis of invasion and metastatic behavior. Zebrafish embryos are transparent, so it is easy to follow the invasion, circulation of tumor cells in blood vessels, migration and micro metastasis formation in real-time (Marques et al., 2009). The entire genome of zebrafish has been determined completely, and the genetic background is clear. Therefore, it can be used for large-scale genetic background screening (Gut et al., 2017). Zebrafish are highly reproductive, with a pair of zebrafish typically producing around 200 embryos (Hoo et al., 2016), and there is little difference between individuals, so they can be used for mass drug screening, such as anti-metastasis drugs (Nakayama et al., 2021). However, the most obvious shortcoming of zebrafish is that it is not a mammal. It is significantly different from humans directly and cannot fully simulate the type of human disease. In addition, antibodies against zebrafish protein are still lacking on the market compared to mice or humans (Hason and Bartùnìk, 2019). In the process of zebrafish xenograft, the temperature of embryo incubation, the different sites for implantation of tumor cells, the interaction between cells and host in embryo, and the changes of tumor microenvironment all affect the experimental results of cell proliferation, invasion and metastasis (Cabezas-Sáinz et al., 2020). Therefore, the technology of xenograft still needs to be improved in different aspects.

### CRISPR/Cas9 for Metastasis Research of Pancreatic Ductal Adenocarcinoma

CRISPR/Cas9 research technology has been developing in recent years and has become a versatile tool for making changes to the genome of many organisms. Here describes some research on CRISPR technology in PDAC metastasis. It has been reported that Mucin 16 (MUC16) contributes to the metastasis of PDAC through FAK mediated Akt and ERK/MAPK pathway. MUC16 knock-out cells generated by CRISPR/Cas9 also exhibit reduced mesenchymal expression and enhanced epithelial expression in PDAC cells and inhibit cell metastasis (Muniyan et al., 2016). Vorvis et al. merged genetic and microRNA profiling analysis with CRISPR/Cas9 technology and identified that transcription factor Forkhead box A2 (FOXA2) is implicated in PDAC pathogenesis. Furthermore, suppression of FOXA2 levels by CRISPR/Cas9 *in vitro* resulted in the activation of the Plasminogen activator, urokinase receptor gene known to be implicated in invasive malignancy. These results were consistent when FOXA2 expression was blocked by siRNA (Vorvis et al., 2016). Core 1 synthase, glycoprotein-N-acetylgalactosamine 3-beta-galactosyltransferase 1 was disrupted in human PDAC cells (T3M4 and CD18/HPAF) by CRISPR/Cas9 leading to enhanced O-glycosylation truncation on MUC16, which increases the formation of aggressive PDACs and metastasis in KPC mice. Stock, al. generated two cortactin knockout lines (PANC-1 and BxPC-3) using CRISPR/Cas9 technology to study the functional role of cortactin in PDAC (Chugh et al., 2018). In PDAC metastases, they detected more expression of cortactin and Tyr421-phosphorylation than the original tumor. Cortactin activation and the migratory ability of the PDAC cells both decreased significantly after treatment with an inhibitor of the Src family kinase. CRISPR/Cas9 technology is also applied to PDAC modeling and therapeutic research using a variety of animals. An individual mouse strain expressing Cas9 in the adult pancreas under a p48 promoter has been established to generate PDAC GEMMs of complicated genotypes with high efficiency (Ideno et al., 2019). By the use of an adeno-associated virus to transfer multiplexed RNA guidelines (sgRNAs) to an adult pancreas of p48-Cre; LSL-Cas9 mice, they produce a mutated Kras G12D allele using homology directed repair in combination with CRISPR-induced disruption of cooperative alleles [Trp53, AT-rich interaction domain 1A (Arid1A) and Lkb1].

Overall, CRISPR-Cas9 engineering provides new opportunities to model PDAC development. It allows the production of syngeneic and humanized mice which can help to eliminate transplant rejection by the host immune system without needing immunocompromised animals (Krempley and Yu, 2017). As a result, these models are particularly useful for researching immunotherapies, allowing to investigate several unanswered problems. For instance, it would be compelling to see if tumors with distinct histopathological features reported in the model have substantial variations in target allele frequency and/or if further mutations have accumulated and how this model’s total mutational and neoantigen load correlates to other GEMMs and human PDAC (Jørgensen and Hogg, 2016).

## Discussion

**FIGURE 2:**
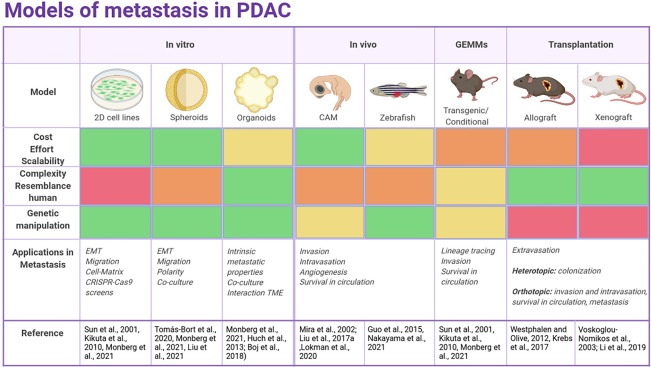
Overview of the current models to study metastasis in PDAC. The color scale indicates whether a model is more suitable (green) or less (red) for each purpose.

Several preclinical models for PDAC are accessible for basic and translational studies, which permit the description of the global genetic features of this disease (summarized in Figure 2).

Pancreatic ductal adenocarcinoma is characterized by an early and fast metastatic process partially related to the location of the pancreas leading to peritoneal, liver or lung metastasis. Nonetheless, a deep molecular insight into the metastatic process of PDAC is still missing since very few studies have studied the mechanisms behind PDAC metastatic organotropism. This is pivotal since the location of the metastases determines the clinical outcome of the patient. Each PDAC preclinical model described above (2D cell lines, spheroids or organoids, GEMMs, and PDXs models) has pros and cons, and the model of choice will vary according to experimental goals.

The correct combination of currently available models is necessary for the development of more trustworthy therapeutic strategies against PDAC. A good strategy is a combination of the methods in each step of the experimental process. Namely, 2D cell lines, PDCL or spheroids are good tools for HTS, studying tumorigenesis and progression. Organoids offer similar benefits as cell lines, adding a step closer to personalized medicine. *In vivo* models are useful to model the TME and immune response, key players in PDAC transformation and progression. Murine models are ideal platforms for understanding PDAC progression, pathophysiology and testing therapeutic modalities. Once candidates are selected, more relevant models like ODXs or PDXs models are suitable for functional validation.

## Author Contributions

MM wrote the abstract, introduction, some models sections, discussion, and elaborated the figures of the manuscript. SZ wrote some models sections, elaborated some tables, and revised the manuscript. CP revised the manuscript, provided critical feedback, and helped shape the manuscript. All authors contributed to manuscript revision, read, and approved the submitted version.

## Conflict of Interest

The authors declare that the research was conducted in the absence of any commercial or financial relationships that could be construed as a potential conflict of interest.

## Publisher’s Note

All claims expressed in this article are solely those of the authors and do not necessarily represent those of their affiliated organizations, or those of the publisher, the editors and the reviewers. Any product that may be evaluated in this article, or claim that may be made by its manufacturer, is not guaranteed or endorsed by the publisher.
